# *Arthrospira platensis* nanoparticles dietary supplementation improves growth performance, steroid hormone balance, and reproductive productivity of Nile tilapia (*Oreochromis niloticus*) broodstock

**DOI:** 10.1371/journal.pone.0299480

**Published:** 2024-06-25

**Authors:** Mohamed M. Mabrouk, Mohamed Ashour, Elsayed M. Younis, Abdel-Wahab A. Abdel-Warith, Mohamed A. Bauomi, Mohamed M. Toutou, Ahmed I. A. Mansour, Basem S. Abdelaty, Mohamed A. Elokaby, Simon J. Davies, Ehab El-Haroun, Ahmed G. A. Gwida

**Affiliations:** 1 Faculty of Agriculture in Cairo, Department of Fish Production, Al-Azhar University, Cairo, Egypt; 2 National Institute of Oceanography and Fisheries (NIOF), Cairo, Egypt; 3 Department of Zoology, College of Science, King Saudi University, Riyadh, Saudi Arabia; 4 Carna Research Station, Ryan Institute, Aquaculture Nutrition Research Unit ANRU, College of Science and Engineering, University of Galway, Galway, Ireland; 5 Faculty of Agriculture, Animal Production Department, Fish Nutrition Research Laboratory, Cairo University, Cairo, Egypt; Tanta University Faculty of Agriculture, EGYPT

## Abstract

This study evaluates the impact of dietary supplementation of the blue-green alga *Arthrospira platensis* NIOF17/003 nanoparticles (AN) on the growth performance, whole-body biochemical compositions, blood biochemistry, steroid hormonal, and fry production efficiency of Nile tilapia (*Oreochromis niloticus*) broodstock, during the spawning season. After a 21-day preparation period to equip the females and ensure that their ovaries were filled with eggs, mating between the mature females and males took place in a 3:1 ratio during a 14-day spawning cycle. A total of 384 tilapia broodstock 288 females and 96 males with an initial body weight of 450.53±0.75, were divided into four groups; AN_0_: a basal diet as a control group with no supplementation of *Arthrospira platensis*, and the other three groups (AN_2_, AN_4_, and AN_6_) were diets supplemented with nanoparticles of *A*. *platensis* at levels of 2, 4, and 6 g kg^─1^ diet, respectively. The results found that fish-fed group AN_6_ showed the highest significant differences in weight gain (WG), final weight (FW), feed conversion ratio (FCR), protein efficiency ratio (PER), and feed efficiency ratio (FER). Females fed the AN_6_ diet showed the highest significant fat content. Compared to the AN_0_ group, fish fed on the supplemented diets showed significant improvement (*p < 0*.*05*) in triglyceride, glucose, and aspartate aminotransferase (AST). A gradual increase in AN inclusion level resulted in a gradual increase in the concentrations of luteinizing hormone (LH), and follicle-stimulating hormone (FSH), testosterone, progesterone, and prolactin. The rates (%) of increase in fry production for females fed supplemented diets were 10.5, 18.6, and 32.2% for AN_2_, AN_4_, and AN_6_, respectively, compared to the control group. This work concluded that the inclusion levels of 6 g kg^─1^ of *A*. *platensis* nanoparticles in the diet of Nile tilapia broodstock significantly improved the growth performances, steroid hormone concentrations, and increased the fry production efficiency by 32.2%, respectively. These findings revealed that *A*. platensis nanoparticles resulted in a significantly enhanced female’ reproductive productivity of Nile tilapia broodstock.

## 1. Introduction

Aquaculture development and sustainability are directly influenced by several factors as global environmental, economic, and political issues such as feed ingredient availability, diet cost production, wars, pandemics, water quality, climate change, ocean productivity, plankton communities, productivity, and nutritional values [1,2]. Globally, tilapia culture has experienced a sharp expansion over the past two decades and is farmed in more than 130 countries worldwide [3]. Tilapia is currently the second most important farmed finfish species in the world [4]. Global production of farmed tilapia grew by 3.3% in 2020 to top 6 million tons for the first time, despite the impact of COVID-19. The expansion of tilapia production all over the world is due to its ability to be produced in various aquatic environments, selective breeding, and its potential to replace marine fish products [1,5,6].

Although several factors limit aquaculture development, such as feeding costs, diseases, bad water quality, the low performance of broodstock, and the high mortality rate in seeds [7,8], several strategic approaches have been recently adopted in aquaculture to sustain tilapia production [9]. One such approach is the production of functional feed that contains health promoters and immune stimulants. Functional feed additives have become the main component of any strategy to control disease outbreaks in aquaculture, particularly when opportunistic bacteria are suspected to be a major cause of mortality [10]. Several functional feed additives have been utilized such as binders, algae derivatives, antimicrobials, seaweed extracts, antioxidants, and enzymes, which improve feed and water quality [11–16]. Other feed additives improve animal performance and health such as immunostimulants probiotics, photogenic, and prebiotics [17–21].

Microalgae recognized with its high amount of bioactive materials, which is significantly higher than any other organisms, microalgae are still utilized in many industries such as human food supplements [22,23], aquaculture feed-additives, water-conditioners [8], phytoremediation [24–28], antimicrobial activities [29,30], cosmetics substances [31–33], pharmaceuticals [34], and biodiesel [28,35–37]. Commonly, *Arthrospira* (*Spirulina*), the blue-green algae, have high protein (50–70% of DW), lipids (5–11%), essential fatty acids (AA, EPA, and DHA), pigments (carotenoid and phycocyanin), minerals (Fe and Ca), vitamins (B_12_ and pro-vitamin A), antioxidant activities, and several molecules which have positively stimulate the attractiveness of a fish diets [38–40]. Therefore, *Arthrospira* is the most family produced around the world due to many reasons [13].

Currently, *Arthrospira* species has been significantly utilized as a feed additive resulting in improved growth performance, feed digestibility, body composition, reduced oxidative damage, and enhanced immune system [8,41,42] for many aquatic animals such as Nile tilapia [6,43,44], hybrid red tilapia *(Oreochromis mossambicus× O*. *niloticus)* [45,46], common carp (*Cyprinus carpio*) [47], Indian major carps, catla and rohu [48], grass carp (*Ctenopharyngodon idella*) [49], rainbow trout (*Oncorhynchus mykiss*) [50], Yellow river carp (*Cyprinus carpio*) [51], Asian seabass (*Lates calcarifer*) [52], European seabass (*Dicentrarchus labrax*) *[53]*, red sea bream (*Pagrus major*) [54], Pacific whiteleg shrimp (*Penaeus vannamei*) [55], shrimp (*Fenneropenaeus chinensisv*) [56], black tiger shrimp (*Penaeus monodon*) [57], and green tiger shrimp (*Penaeus semisulcatus*) [55]. There is a positive relationship between dietary microalgae inclusion, especially *Arthrospira*, and the reproductive performance of aquatic animals. As indicated by several studies [58–60], *Arthrospira* sp. supplementation has notable impacts on reproductive performance through its involvement in hormonal regulation, specifically concerning the reproductive system. It enhances fertility, restores the antioxidant status of the ovary, and contributes to ovary signaling. Beresto [61] found that supplementing female minks with Spirulina at doses of 200 and 400 mg/animal resulted in a decrease in the percentage of abortive females, while simultaneously increasing litter size. This finding is consistent with previous research conducted on nanny goats and doe rabbits, which also demonstrated improved litter size with Spirulina supplementation [60]. In another study, Iatrou et al. [62] reported that *Arthrospira*-treated female mink tended to an increased whelping rate.

Besides its applications as aqua feed additives, *Arthrospira* or its derivatives have many biotechnological applications. The lipid-free dry weight (biodiesel byproduct) of *A*. *platensis* NIOF17/003 was successively utilized as dry feed for rotifer (*Brachiounus plicatilis*), in the same line to remove ammonia and organic dye from aquaculture wastewater effluents and industrial textile effluents, respectively [63,64]. The growth of aquaculture has raised the demand for better diets and aqua-feed additives. Recently, several studies have documented different forms of aqua-feed additives in aquaculture feed. The form of feed-additive inclusion is of high importance to maximize the utilization of added materials [65]. Recently, interest in using nanoparticles of several materials as animal feed additives has been expanded attributed to the higher bioavailability and efficiency [57].

Nanotechnology applications have been successfully increased [66–68]. Algae nanoparticle applications in aqua feed diets also increased due to their high of their high surface area of nanoparticles [69] which enhances growth performance, feed utilization, body composition, stress tolerance, and enhanced immune system for many species such as Nile tilapia [42,70], Pacific white shrimp [42], black tiger shrimp [57], and Zebrafish [71]. The current study aims to evaluate the effect of the cyanobacterium species, *Arthrospira platensis* NIOF17/003 nanoparticles, as a functional feed additive on growth performances, whole-body biochemical composition, blood biochemistry, steroid hormonal status, and seeds production efficiency for the Nile tilapia broodstock during the spawning cycle.

## 2. Materials and methods

### 2.1. *Arthrospira platensis* NIOF17/003 nanoparticles

As previously described [8], *Arthrospira* (*Spirulina*) *platensis* NIOF17/003 was isolated from a saline-alkaline lake named El-Khadra Lake located in Wadi El-Natrun, north-west of Egypt, genetically identified, and deposited in the GenBank database with accession number: MW396472. The biomass productivity (143.82 mg L^─1^ day^─1^), lipid productivity (14.37 mg L^─1^ day^─1^), total protein (52.03% of dry weight base), total carbohydrates (14%), total lipids (8.52%), and fatty acid profiles of saturated (42.27%), monounsaturated (26.71%), polyunsaturated (31.04%), and ω-3 (3.16%) fatty acids of *A*. *platensis* NIOF17/003 are determined by [63]. The nanoparticles preparation of *A*. *platensis* was performed, at the Egyptian Petroleum Research Institute (EPRI), Nasr City, Cairo, Egypt, using Ball grinding (Planetary Ball Mill PM 400 “4 grinding stations”) as described in a previous study [8]. Compared to the normal particle size of *A*. *platensis* (with an average of 100 μ mL^─1^), the nanoparticle size of *A*. *platensis* (averaged of 87.6%) revealed a nanoparticle average of 183.9 nm, as reported in our previous studies [8,42]. Moreover, the GC-mass phytochemical analysis was determined as described by our previous studies [8,41]. It was reported that the bioactive compounds found in *A*. *platensis* nanoparticles, which are used in the current study, were found to contain three main bioactive compounds, namely: (1) milbemycin b (C_33_H_46_ClNO_7_), which accounted for 66% of the total peak areas (PAs), (2) Docosanoic acid 1,2,3-propanetriyl ester (C_69_H_134_O_6_), accounting for 22% of the total PAs, and (3) Copper etioporphyrin II (C_32_H_36_CuN_4_), accounting for 11% of the total PAs. These three bioactive compounds belong to different categories (macrocyclic lactones, fatty acid propanetriyl ester, and metal Porphyrin Complex, respectively) and exhibit antioxidant, antimicrobial, and biomedical activities [72].

### 2.2. Water quality indices

Following the guidelines of APHA [73], water quality parameters of ammonia (NH_3_, mg L^─1^), nitrate (NO_3_, mg L^─1^), nitrite (NO_2_, mg L^─1^), and dissolved oxygen (DO, mg L^─1^) were determined three times a week. Furthermore, pH, salinity (ppt), and temperature (°C) were determined daily (1.00 pm) during the experimental period and Table 1 shows the water quality parameter during the experimental period. During the 14-days spawning cycle and the 21-day equipping period, all remarkable water qualities were within the recommended range of production requirements of Nile tilapia broodstock during the spawning season.

**Table 1:** Water quality parameters during the spawning cycle experiment.

Water quality parameters	Experimental diets			
	AN_0_	AN_0_	AN_0_	AN_0_
pH	7.30±0.09	7.42±0.01	7.38±0.13	7.53±0.28
Salinity (ppt)	0.98±0.02^a^	0.90±0.02^b^	0.99±0.02^a^	0.97±0.01^a^
Temperature (°C)	25.84±0.90^a^	26.25±0.87^a^	25.46±0.84^ab^	26.65±0.75^a^
DO (mg L^─1^)	6.62±0.09^ab^	6.57±0.01^b^	6.91±0.13^a^	6.83±0.08^a^
NO_2_ (mg L^─1^)	0.181±0.015^a^	0.183±0.042^a^	0.171±0.042^b^	0.182±0.011^a^
NH_3_, (mg L^─1^)	0.110±0.001	0.107±0.016	0.111±0.015	0.109±0.007
(NO_3_, mg L^─1^)	0.202±0.023	0.203±0.018	0.202±0.005	0.224±0.003

*AN_0_, AN_2_, AN_4_, and AN_6_: Diets supplemented with *A*. *platensis* nanoparticles at levels of 0, 2, 4, and 6 g kg^─1^ diet, respectively. The presented data are Means ± SD (*n = 3*). Different letters in the same raw are significantly different (*p* < 0.05). The absence of letters in the same row means no significant differences.

### 2.3. Nile tilapia (*Oreochromis niloticus*) broodstock

#### 2.3.1. Experimental fish and design

The current experiment was carried out in a private Tilapia hatchery located in Port Said Governorate, Egypt. Nile tilapia broodstock (males and females) was obtained from a commercial farm of Nile tilapia located in Port Said Governorate, Egypt. The fish were given a control-based diet for 21 days before starting the feeding trial to initiate the spawning cycle. After 21 days of acclimation, during which the females’ ovaries were investigated to be ready for spawning, mating took place between males and females in a 3:1 ratio for 14 days. In the current experiment, the main source of water was the irrigation water of the El-Slam Canal, and the rate of daily freshwater change was 30%. Daily, fish faeces, unconsumed feed, and wastes were removed by siphoning. The tanks were aerated using an air blower. The experiment was conducted with four groups, in a greenhouse with 12 concrete tanks of 8 m^3^ (2 m x 4 m x 1 m) each. A total 384 of Nile tilapia broodstock (288 females and 96 males) were divided into four groups, each having three replicates.

#### 2.3.2. Experimental diet

As presented in Table 2, four diets were used in this study: AN_0_: a basal diet as a control group, while the other three groups (AN_2_, AN_4_, and AN_6_) were basal diet supplemented with nanoparticles of *A*. *platensis* at levels of 2, 4, and 6 g kg^─1^ diet, respectively. The addition of respective levels of *A*. *platensis* nanoparticles to diets was performed as previously described by Mabrouk et al. [8]. Briefly, The prepared nanoparticle powder of *A*. *platensis* nanoparticle was dissolved in an adequate volume of distilled water and set aside to be mixed with the remaining diet ingredients. The diet ingredients were formulated by thoroughly combining and the A. platensis nanoparticles were sprayed on four sets of the experimental diets at the rates of 0, 2, 4, and 6 g/kg diet. Following this, the oil and water were mixed extensively with the ingredients, and the mixture was pelleted using the Sprout-Waldron Laboratory Pellet Mill (CPM, California Pellet Mill Co., USA) to create 2 mm pellets. The pellets were then dried in ovens at 40°C until the moisture level dropped below 10% [8]. The biochemical composition of basal diet, based on the % of dry matter bases, of crude protein (29.9%), ether extract (9.2%), crude fiber (4.7%), nitrogen-free extract (48.7%), ash (7.5%), gross energy (4963 kj kg^─1^ diet), and digestible energy (3520 kj kg^─1^ diet) were calculated according to the reported guideline of AOAC [74]. Fish were hand-fed three times daily, at 9 am, 12 pm, and 4 pm, at a rate equivalent to 3% of their wet body weight.

**Table 2:** Composition analysis (%) of the experimental diets.

Diets Composition and analysis	Experimental diets			
	AN_0_	AN_2_	AN_4_	AN_6_
Composition (% of dry weight)*				
Fish meal	14	14	14	14
Soybean meal	20	20	20	20
Yellow corn	25	25	25	25
Rice bran	15	15	15	15
Wheat bran	15	15	15	15
Corn gluten	7	7	7	7
Soya oil	3	3	3	3
Dicalcium phosphate	0.7	0.7	0.7	0.7
Premix Mixer	0.3	0.3	0.3	0.3
Total	100	100	100	100
*Arthrospira platensis* nanoparticles supplementation levels (g kg^─1^)	0	2	4	6

*AN_0_, AN_2_, AN_4_, and AN_6_: Diets supplemented with *Arthrospira platensis* nanoparticles at levels of 0, 2, 4, and 6 g kg^─1^ diet, respectively. Each 1 kg premix contains (mg kg^─1^): P-amino benzoic acid (9.48), D-biotin (0.38), inositol (379.20), niacin (37.92); Ca-pantothenate (56.88), Pyridoxine-HCl (11.38), riboflavin (7.58), Thiamine-HCl (3.79), L-ascorbyl-2-phosphate Mg (296.00), folic acid (0.76), cyanocobalamin (0.08), menadione (3.80), vitamin A-palmitate (17.85), a-tocopherol (18.96), calciferol (1.14), K_2_PO_4_ (2.011), Ca_3_ (PO_4_)_2_ (2.736), Mg SO_4_.7H_2_O (3.058), and NaH2PO_4_.2H_2_O (0.795).

### 2.4. Tested parameters

#### 2.4.1. Growth indices

At the end of the rearing trial, fish were starved for 24 h to empty the digestive tract [75]. After that, the total body weight and the total number of each replicate were investigated to calculate weight gain (WG), specific growth rate (SGR), feed conversion ratio (FCR), protein efficiency ratio (PER), and survival rate (SR) using the given formulas.

To calculate weight gain (WG), the initial weight (IW, 450.53 ± 0.75) and final weight (FW) of mothers were determined before and after the 14-days spawning cycle experiment. No mortality was observed during the experiment. Moreover, the indices of feed conversion ratio (FCR), protein intake (PI), protein efficiency ratio (PER), and feed efficiency ratio (FER) were calculated as described below.


$$\begin{array}{c} WG\;(g)=FBW–IBW\\ where,\;FBW\;and\;IBW\;are\;the\;final\;and\;initial\;body\;weight\;(g),\;respectively. \end{array}$$ (1)

$$\begin{array}{c} SGR\;(\%,\;day)=100\times (In\;FBW–In\;IBW)/t\\ where,\;ln=natural\;logarithmic;\;and\;t=time\;in\;days. \end{array}$$ (2)

Feedconversionratio(FCR)=Dryweightoffeedconsumed(g)/weightgain(g) (3)

Proteinefficiencyratio(PER)=Weightgain(g)/Proteinfed(g) (4)

SR(%)=100×(FinalNumber/InitialNumber) (5)

#### 2.4.2. Biochemical analysis

At the end of a 14-day spawning cycle experiment, fish in all dietary treatments were starved for 24 h, and five fish (three females and two males) were randomly collected, homogenized, dried, ground, and stored under -20°C for whole-body analysis as described elsewhere [41]. The whole-body biochemical composition of fish and diets were determined, and moisture, dry matter (DM), crude protein (CP), ether extract (EE), crude fiber (CF), nitrogen-free extract (NFE), gross energy (GE), and digestible energy (DE) were determined and calculated according to the guideline of AOAC [74].

#### 2.4.3. Blood serum analysis

At the end of the experiment, six fish samples (three males and three females) from each replicate were anesthetized using TMS buffered (Tricaine Methanesulfonate at the dose of 30 mg L^─1^) to collect blood serum for blood biochemistry analysis protocol as previously described by Ferguson et al. [76]. Blood samples were extracted using a sterilized hypodermic syringe (3 mL with a 22-gauge needle and a heparinized tube) and stored at room temperature for 30 minutes and then centrifuged at 3.000 RPM for 15 m. The collected serum was stored at -20°C for further analysis. The concentration of total protein (g dL^─1^) [77] and albumin (g dL^─1^) [78] were measured, and the globulin level (g dL^─1^) was calculated as the difference between the values of total protein and albumin. The levels of glucose (mg dL^─1^) [79] and triglyceride (TAG) (mg dL^─1^) [80] levels were measured using kits from El-Nasr Pharmaceutical Chemicals Co., Egypt, following the provided instructions. Moreover, using the calorimetric techniques, the activities of serum glutamic pyruvate transaminase (GPT, U mL^─1^) [81]. Aspartate aminotransferase (AST, U mL^─1^), and alanine (ALT, U mL^─1^) were determined according to [82] using commercial kits from Biodiagnostic in Egypt, as per the manufacturer’s guidelines.

#### 2.4.4. Steroid hormones (SHs)

At the end of the spawning trial, from each replicate, six mothers’ samples (three males and three females) were randomly selected to determine SHs. For both males and females, luteinizing hormone (LH), and follicle-stimulating hormone (FSH) were determined. For males only, total testosterone (TT) and free testosterone (FT) were determined. For females only, prolactin and progesterone hormones were determined. The SHs were calorimetry determined using ELISA assay as described elsewhere [83], an enzyme-linked immune sorbent, known as the Immulite/Immulite 1000 system [84]. The fish-specific commercial kits of FSH (RAB0660-1KT), LH (SE120071), TT (SE120120), FT (SE120120), PRO (RAB0408-1KT), and PRG (SE120087) were determined, following to the manufacturer’s instructions.

#### 2.4.5. Females reproductive productivity

After the 14-days spawning trial, the adult fish were carefully gathered and transferred to different ponds, while lowering the water level. The method described by El-Sayed et al. [85] was followed to collect the eggs. The number of offspring produced by each female was calculated using the following formula:

$$Number\;of\;fries\;per\;female=\frac{Total\;number\;of\;seeds/pond}{\;Total\;number\;of\;females/pond}$$ (6)

The average ratio (%) of the number of seeds from mothers fed the control diet to the supplemented diets was conducted as the following Eq.:

$$Number\;of\;seeds\;per\;female\;=\frac{Sn-Sc}{Sc}\times 100$$ (7)

Where Sn and Sc are the numbers of seeds that come from mothers fed the supplemented and the control diets, respectively.

### 2.5. Statistical analysis

The data were assessed for homoscedasticity and normality before conducting statistical analysis. The results were presented as mean ± standard deviation (n = 3). The statistical analysis was performed using the SPSS computer software package. To determine significant differences among means at a p-value of less than 0.05, a one-way ANOVA was performed followed by Duncan’s multiple-range tests. The graphical representation of the steroid hormone levels and broodstock seed production figures was created using GraphPad Prism version 9.

## 3. Results

### 3.1. Growth indices

The growth performances of Nile tilapia, during the spawning cycle, are presented in Table 3. Significant differences (*p < 0*.*05*) were revealed in FW, WG, FCR, PER, and FER between the control group (AN_0_) and the groups supplemented with *A*. *platensis* nanoparticles (AN_2_, AN_4_, and AN_6_). However, fish-fed group AN_6_ showed the highest significant differences (*p < 0*.*05*) in FW, WG, FCR, PER, and FER.

**Table 3:** Growth performance indices of *O*. *niloticus* broodstock during the spawning season.

Growth performance indices	Experimental diets			
	AN_0_	AN_2_	AN_4_	AN_6_
IW (g)	450.53±0.75	450.47±0.80	450.07±0.42	450.03±0.21
FW (g)	456.00±0.36^b^	455.87±0.35^b^	456.10±0.87^b^	458.69±0.42^a^
WG (g)	5.47±0.58^b^	5.41±1.06^b^	6.80±1.01^ab^	8.65±1.30^a^
FCR	1.97±0.05^a^	1.99±0.02^a^	1.78±0.06^a^	1.41±0.21^b^
PI (g)	3.29±0.34	3.28±0.65	3.58±0.69	3.58±0.08
PER	0.51±0.012^b^	0.50±0.006^b^	0.56±0.021^b^	0.72±0.101^a^
FER	1.66±0.042^b^	1.65±0.015^b^	1.84±0.057^b^	2.35±0.321^a^

* AN_0_, AN_2_, AN_4_, and AN_6_: Diets supplemented with *A*. *platensis* nanoparticles at levels of 0, 2, 4, and 6 g kg^─1^ diet, respectively. The presented data are Means ± SD (*n = 3*). Different letters in the same raw are significantly different (*p* < 0.05). The absence of letters in the same row means no significant differences (*p* < 0.05). WG: Weight gain, FW: Final weight, IW: Initial weight, FCR: Feed conversion ratio, PI: Protein intake, PER: efficiency ratio, and FER: Feed efficiency ratio.

### 3.2. Whole-body proximate composition

**Fig 1:**
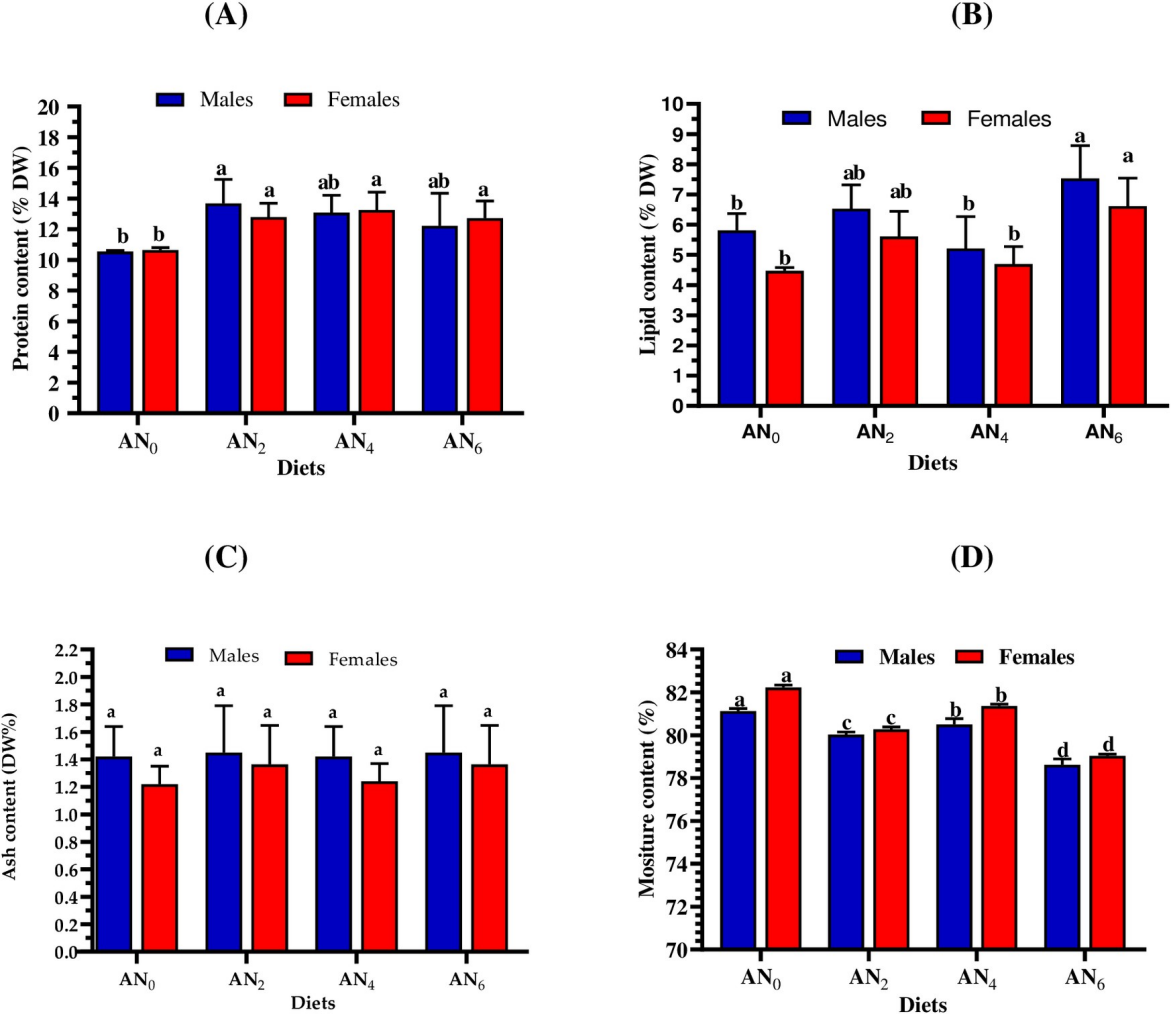
Whole body analysis contents of protein (A), lipid (B), ash (C), and moisture (D) of *O*. *niloticus* (males and females) fed different inclusion levels of *A*. *platensis* nanoparticles. AN_0_, AN _2_, AN_4_, and AN_6_ are diets supplemented with *A*. *platensis* nanoparticles at levels of 0, 2, 4, and 6 g kg^─1^ diet, respectively. The presented data are Means ± SD (*n = 3*). Different letters in each column are significantly different (*p < 0*.*05*).

Fig 1 shows the approximate whole-body compositions (protein, lipid, ash, and moisture) of Nile tilapia fed the control diets and the diets supplemented with *A*. *platensis* nanoparticles. Compared to the control group, both males and females fed with supplemented diets (AN_2_, AN_4_, and AN_6_) achieved the highest protein content (Fig 1A). The highest significant (*p* < 0.05) lipid content was observed by fish fed the group of AN_6_, followed by AN_2_, AN_4_, and AN_0_ (Fig 1B). Among the dietary groups, no significant differences (*p < 0*.*05*) were observed in ash content (Fig 1C), and the lowest moisture content was observed by mothers fed the control group (AN_0_) (Fig 1D).

### 3.4. Blood biochemistry

Table 4 shows the blood serum biochemical analysis of Nile tilapia fed different inclusion levels of *A*. *platensis* nanoparticles. Among the dietary groups, no significant differences (*p < 0*.*05*) were observed in the total protein, albumin, globulin, GPT, and ALT contents. Compared to the control group, fish feed *A*. *platensis* nanoparticles (AN_2_, AN_4_, and AN_6_) showed significant differences (*p < 0*.*05*) in TAG, glucose, and AST. The highest significant (*p < 0*.*05*) TAG was observed in fish fed the AN_4_ diet, followed by AN_6_, and AN_2_, while the lowest TAG content was observed in control fish. The highest significant (*p < 0*.*05*) glucose was observed in fish fed with AN_6_, followed by AN_4_, and AN_2_, while the lowest glucose was revealed in fish fed the control diet. On the other hand, compared to the *A*. *platensis* nanoparticles groups, control-fed fish showed significantly (*p < 0*.*05*) the highest AST content, followed by AN_2,_ AN_4_, and AN_6_.

**Table 4:** Blood biochemical parameters of *O*. *niloticus* broodstock fed different inclusion levels of *A*. *platensis* nanoparticles.

Blood parameters	Experimental diets			
	AN_0_	AN_0_	AN_0_	AN_0_
Total Protein (g dL^─1^)	6.20 ± 0.36	6.03 ± 0.57	5.93 ± 0.93	6.07 ± 0.15
Albumin (g dL^─1^)	4.47 ± 0.67	4.27 ± 0.15	3.93 ± 0.67	4.20 ± 0.46
Globulin (g dL^─1^)	1.73 ± 0.35	1.77 ± 0.65	2.00 ± 0.26	1.87 ± 0.31
Triglyceride (mg dL^─1^)	191.33 ± 7.57^b^	203.37 ± 5.51^ab^	211.33 ± 14.57^a^	203.67 ± 6.03^ab^
Glucose (mg dL^─1^)	104.33 ± 5.03 ^b^	109.33 ± 5.51^ab^	116.00 ± 7.00^ab^	119.67 ± 7.51^a^
GPT (U mL^─1^)	27.33 ± 5.86	28.67 ± 5.69	25.67 ± 2.52	29.00 ± 5.29
AST (U mL^─1^)	68.6 ± 2.08^a^	64.30 ± 2.23^b^	58.61 ± 3.46^b^	56.43 ± 2.31^b^
ALT (U mL^─1^)	27.32 ± 3.38	28.71 ± 3.28	25.72 ± 1.45	29.17 ± 3.05

AN_0_, AN_2_, AN_4_, and AN_6_ are diets supplemented with *A*. *platensis* nanoparticles at levels of 0, 2, 4, and 6 g kg^─1^ diet, respectively. GPT: Glutamic pyruvate transaminase (U mL^─1^), AST: Aspartate aminotransferase (U mL^─1^), ALT Alanine (U mL^─1^). The presented data are Means ± SD (*n = 3*). Different letters in the same row are significantly different (*p < 0*.*05*), while the absence of letters means no significant differences.

### 3.5. Steroid hormones

**Fig 2:**
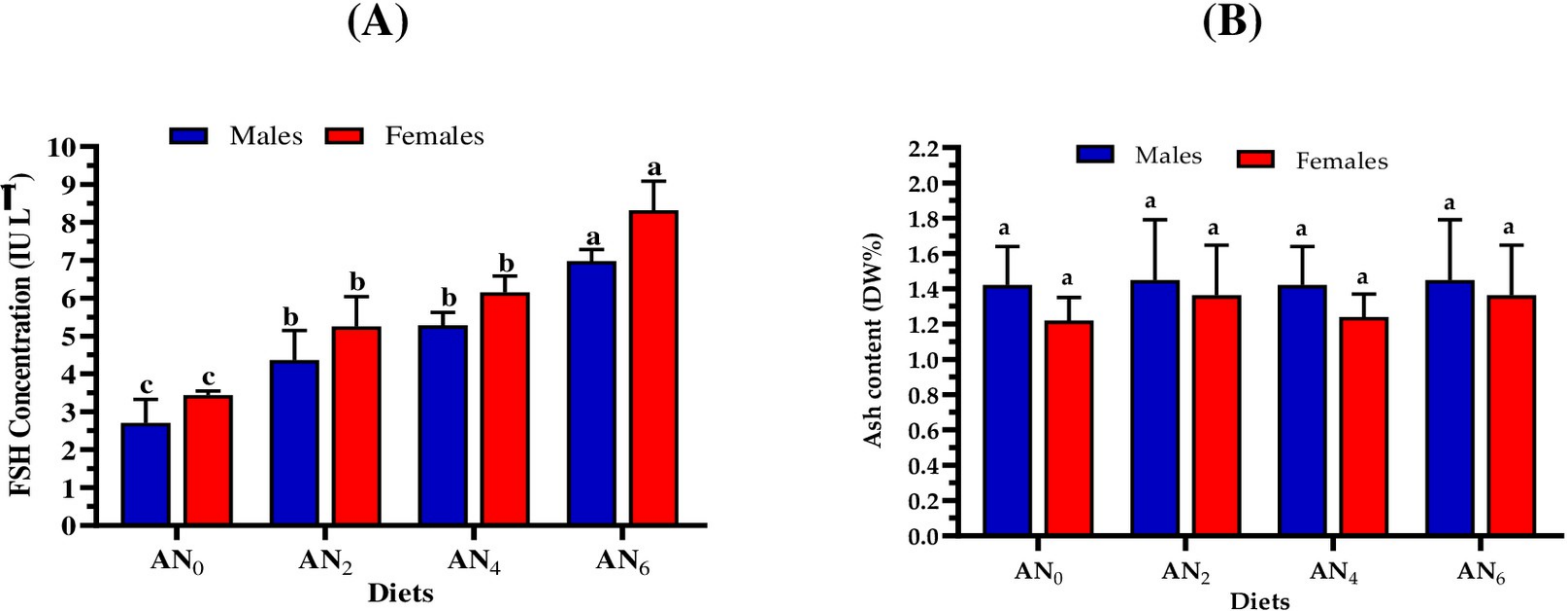
Impact of different *A*. *platensis* nanoparticles inclusion levels on the (A) follicle-stimulating hormone, FSH, and (B) luteinizing hormone, LH, of *O*. *niloticus* broodstock (males and females). AN_0_, AN_2_, AN_4_, and AN_6_ are diets supplemented with *A*. *platensis* nanoparticles at levels of 0, 2, 4, and 6 g kg^─1^ diet, respectively. The presented data are Means ± SD (*n = 3*). Different letters in each column are significantly different (*p < 0*.*05*).

**Fig 3:**
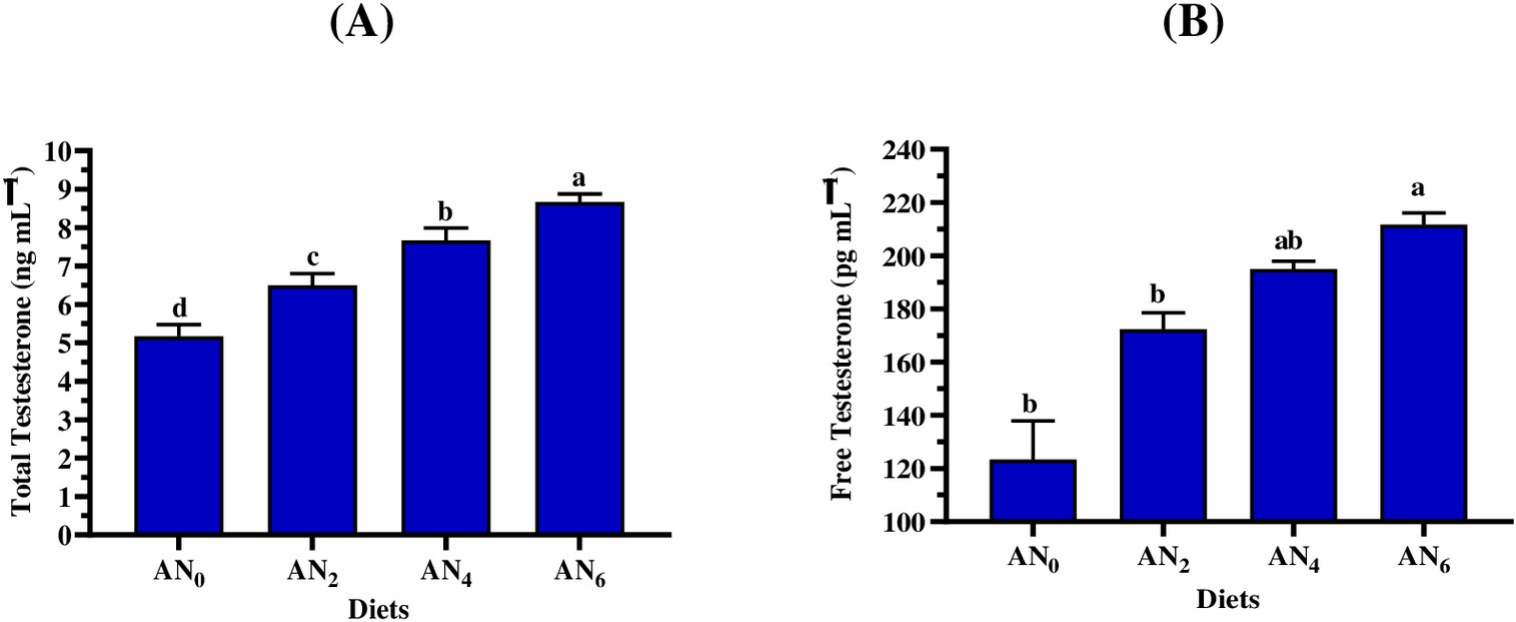
Impact of different *A*. *platensis* nanoparticles inclusion levels on the (A) total testosterone, and (B) free testosterone of *O*. *niloticus* broodstock (males only) of *O*. *niloticus*. AN_0_, AN_2_, AN_4_, and AN_6_ are diets supplemented with *A*. *platensis* nanoparticles at levels of 0, 2, 4, and 6 g kg^─1^ diet, respectively. The presented data are Means ± SD (*n = 3*). Different letters are significantly different (*p < 0*.*05*).

**Fig 4:**
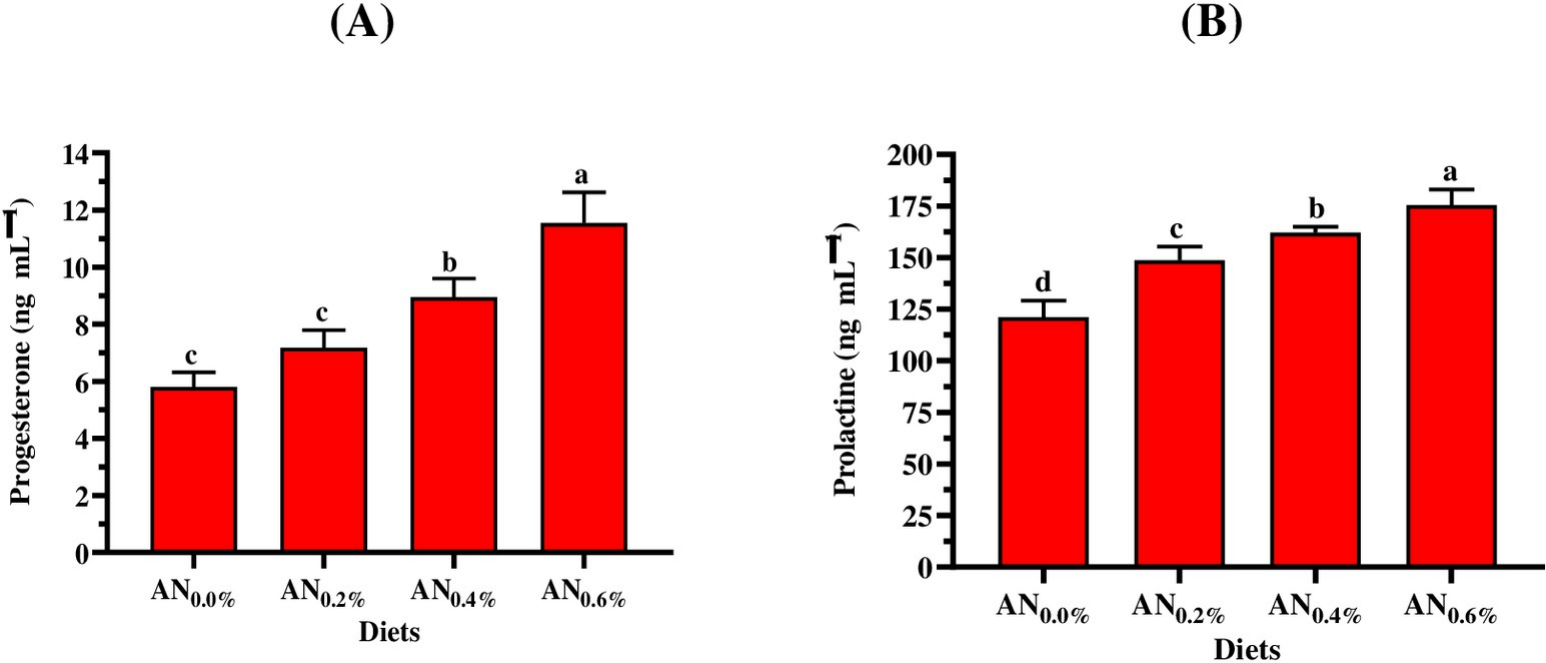
Impact of different *A*. *platensis* nanoparticles inclusion levels on the (A) progesterone, and (B) prolactin of *O*. *niloticus* (females only) of *O*. *niloticus*. AN_0_, AN_2_, AN_4_, and AN_6_ are diets supplemented with *A*. *platensis* nanoparticles at levels of 0, 2, 4, and 6 g kg^─1^ diet, respectively. The presented data are Means ± SD (*n = 3*). Different letters are significantly different (*p < 0*.*05*).

Figs 2–4 show the influence of different inclusion levels of *A*. *platensis* nanoparticles on steroid hormone concentrations in Nile tilapia males and females. Fig 2 displayed that fish-fed supplemented diets revealed a significant (*p < 0*.*05*) improvement in FSH and LH concentrations in both females and males, compared to control-fed fish. Moreover, a gradual increase in incorporation levels of *A*. *platensis* nanoparticles resulted in a gradual increase in LH and FSH concentrations. Furthermore, the females showed a positive response to the gradual increase in inclusion levels higher than males (Fig 2). Fig 3 shows that the gradual increase in inclusion levels of *A*. *platensis* nanoparticles resulted in a gradual increase in the concentrations of testosterone (total and free). Compared to the control group, significant (*p < 0*.*05*) improvements in total and free testosterone concentrations were obtained by males fed diets supplemented with *A*. *platensis* nanoparticles (AN_2_, AN_4_, and AN_6_) and (AN_4_ and AN_6_), respectively. Fig 4 shows that the gradual increase in inclusion levels of *A*. *platensis* nanoparticles led to a gradual increase in the concentrations of progesterone prolactin hormones. Compared to the control group, significant (*p* < 0.05) improvements in progesterone and prolactin concentration were obtained by males who consumed the supplemented diets (AN_4_ and AN_6_) and (AN_2_, AN_4_, and AN_6_), respectively.

### 3.6. Females’ reproductive productivity

**Fig 5:**
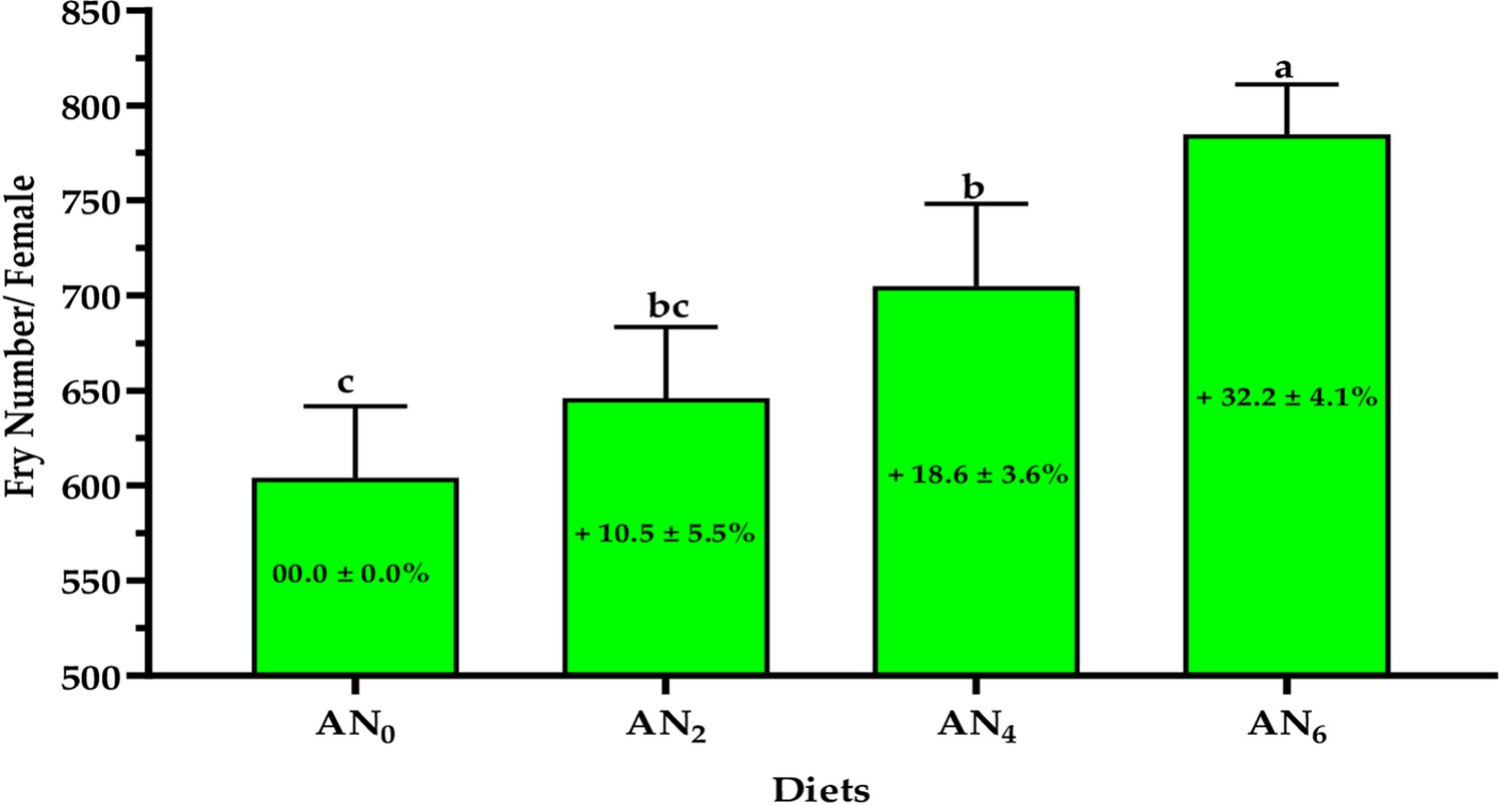
Impact of different nanoparticles inclusion levels of *A*. *platensis* on the seed production efficiency of *O*. *niloticus*. AN_0%_, AN_2_, AN_4_, and AN_6_ are diets supplemented with *A*. *platensis* nanoparticles at levels of 0, 2, 4, and 6 g kg^─1^ diet, respectively. The presented data are Means ± SD (*n = 3*). Different letters are significantly different (*p < 0*.*05*). The percentages in the bars are the increased percentage (%) in seed production for females fed supplemented diets compared to the control diet.

Fig 5 shows the impact of different inclusion levels of *A*. *platensis* nanoparticles on the seed’s production count. A gradual increase in incorporation levels resulted in a significant (*p < 0*.*05*) gradual increase in seed production efficiency. Compared to control-fed fish, increasing rates in seed production were noticed for AN_2_, AN_4_, and AN_6_ diets, 10.5, 18.6, and 32.2%, respectively.

## 4. Discussion

According to the literature, algal cells (microalgae or seaweeds) and bioactive compounds extracted from algal such as astaxanthin from *A*. *platensis* [41], and polysaccharides extracted from brown seaweed (*Sargassum dentifolium*) [8,42,86] were included as feed additives in various forms as dry powder, liquid extract [65]. The nanoparticle form is the newest technology that can improve diet efficiency [70]. A study conducted by Nagarajan et al. [87] reported that these positive effects of nanoparticle forms may be due to the novel properties of highly fine particles and the high surface area of the molecules. These new properties can change, maximize, and create novel properties for the phytochemical compounds in the microalgae nanoparticle forms compared to the traditional microalgae form [88–91].

In previous studies, the bioactive compounds of *A*. *platensis* nanoparticles used in the present study were reported [8,41]. Three peak areas (PAs) were found in *A*. *platensis* nanoparticles. These three PAs were found to contain three main bioactive compounds, namely: (1) milbemycin b (C_33_H_46_ClNO_7_), which accounted for 66% of the total peak areas (PAs), (2) Docosanoic acid 1,2,3-propanetriyl ester (C_69_H_134_O_6_), accounting for 22% of the total PAs, and (3) Copper etioporphyrin II (C_32_H_36_CuN_4_), accounting for 11% of the total PAs. These three bioactive compounds belong to different categories (macrocyclic lactones, fatty acid propanetriyl ester, and metal Porphyrin Complex, respectively) and exhibit antioxidant, antimicrobial, and biomedical activities [72,92].

To the best of our knowledge, no study has investigated the effects of nanoparticles of microalga *A*. *platensis* on Nile tilapia during the spawning period. The present study showed improvement in growth performances, whole-body biochemical composition, physiological aspects, steroid hormonal status, and fry production efficiency for the Nile tilapia during the spawning cycle. The current study revealed that the AN_6_ group has achieved the highest significant differences (*p < 0*.*05*) in FW, WG, FCR, PER, and FER, compared to the control group (AN_0_) and the other supplemented groups (AN_2_ and AN_4_). Elabd et al. [70] revealed that inclusion levels of 2.5–5 g kg^─1^ of *A*. *platensis* nanoparticles into Nile tilapia diets significantly improved growth performance indices. In a later study, Sharawy et al. [42] reported that the inclusion of *A*. *platensis* nanoparticles (2.5, 5, and 10 g kg^─1^ diet) in a Pacific white shrimp diet significantly (*p < 0*.*05*) improved the growth performances of shrimp fry. The findings reported in the present study reported that the inclusion concentrations (6 g kg^─1^ diet) achieved an economic advantages regarding the growth performance. However, this small increase in FW and WF may be attributed to the fact that the broodstock at this age tends to direct all its energy to the steroid hormonal aspects, ovulation, and egg production, not to the growth and building tissues.

Aquafeeds are significantly affecting the carcass composition of aquatic animals, especially in early growth stages [93,94]. In the current study, supplemented groups significantly affected the protein and the lipid content of Nile tilapia mothers. These results are in accordance with the previous studies which concluded that the nanoparticles form of *A*. *platensis* inclusion levels to Nile tilapia significantly improves protein and lipid contents [8,42,70]. This finding may be attributed to the fact that *A*. *platensis* is a rich source of protein (50–65%) and has a high-quality fatty acid profile (AA, EPA, and DHA) with total lipid content of 4–8% [39].

Blood biochemistry indices (serum protein, albumin, TAG, glucose, GPT, AST, and ALT) are major factors in improving blood aspects, immune system, and overall physiological status of fish and act powerfully as adjuncts to assess the efficiency of feed additives [95–97]. The current study reported that the only significant difference in blood biochemistry indices was recorded in TAG, and glucose by the supplemented diets compared to the control diet. These results are in accordance with previous results of Mabrouk et al. [8], Elabd et al. [70], and Sharawy et al. [42]. However, these results may be due to the high- lipid content of *A*. *platensis* nanoparticles dietary supplementations [98].

To achieve successful and effective reproduction of aquatic animals, it’s essential to understand the relationship between hormonal spawning and nutritional and environmental factors such as diet development, photoperiod, and temperature which sequentially, affect seed production efficiency [99,100]. In the current study, different nanoparticle inclusion levels of *A*. *platensis* significantly (*p < 0*.*05*) affect hormonal spawning for Nile tilapia broodstock (males and females). The current study observed that a gradual increase in incorporation nanoparticle levels resulted in a gradual increase in hormonal spawning (FSH, LH, free testosterone, total testosterone, progesterone, and prolactin). Compared to the control group (AN_0_), increasing rates in fry production for the supplemented diets of AN_2_, AN_4_, and AN_6_ were 10.5, 18.6, and 32.2%, respectively were revealed. These findings were in accordance with the results of several studies which concluded that *Arthrospira* (*Spirulina*) strains can improve the formation of ovulation, prostaglandin, and steroidogenesis, improve maturation ability, optimize the levels of sex hormones, enhance reproduction performance and hatching efficiency, and increase seeds production in fish species such as Nile tilapia [101], catfish (*Clarias gariepinus*) [102], zebrafish females [103], three-spot gourami (*Trichopodus trichopterus*) [104], yellow tail cichlid (*Pseudotropheus acei*) [105], parrot fish (*Oplegnathus fasciatus*) [106], Pla Pho (*Pangasius bocourti*) [107], and goldfish (*Carassius auratus*) [108]. Promya and Chitmanat [102] concluded that *Arthrospira* is an alternative candidate to artificial hormones in the diet of fish brooders. Interestingly, due to their novel physical properties and bioactive compounds, the nanoparticle form of *A*. *platensis* maximizes the nutritional benefit for Nile tilapia.

Joshua and Zulperi [98] reported that the nutritional impact and bioactive material contents of *A*. *platensis* and *Chlorella vulgaris*, they are algal species that can significantly enhance the immune system, reduce disease infections, improve the hormonal spawning, and improve reproduction aspects of fish and shrimp. Hassaan [109] concluded that the inclusion of 10–15 g kg^─1^ of microalgae *Cyclotella* spp. (dried form) in the diet of Nile tilapia broodstock significantly improved hormonal spawning, gonadosomatic index, condition factor, semen quality, and relative absolute fecundity, which consequentially improved seed production.

## 5. Conclusions

Inclusion levels of 6 g kg^─1^ of *A*. *platensis* nanoparticles in the diet of Nile tilapia broodstock significantly improved the growth performances (FW, WG, FCR, PER, and FER), steroid hormones levels (FSH, LH, free testosterone, total testosterone, progesterone, and prolactin), and increase fry production efficiency of 32.2%, respectively. These findings revealed that *A*. *platensis* nanoparticles resulted in a better enhancement of females’ reproductive productivity of Nile tilapia.
