# Correction: *Arthrospira platensis* nanoparticles dietary supplementation improves growth performance, steroid hormone balance, and reproductive productivity of Nile tilapia (*Oreochromis niloticus*) broodstock

**DOI:** 10.1371/journal.pone.0334463

**Published:** 2025-10-13

**Authors:** Mohamed M. Mabrouk, Mohamed Ashour, Elsayed M. Younis, Abdel-Wahab A. Abdel-Warith, Mohamed A. Bauomi, Mohamed M. Toutou, Ahmed I. A. Mansour, Basem S. Abdelaty, Mohamed A. Elokaby, Simon J. Davies, Ehab El-Haroun, Ahmed G. A. Gwida

In Table 4, all treatments are labelled as “AN_0_” instead of “AN_0_, AN_2_, AN_4_ and AN_6_”. Please see the correct Table 4 here.

**Table 4:** Blood biochemical parameters of *O. niloticus* broodstock fed different inclusion levels of *A. platensis* nanoparticles.

Blood parameters	Experimental diets			
	AN_0_	AN_2_	AN_4_	AN_6_
Total Protein (g dL^─1^)	6.20 ± 0.36	6.03 ± 0.57	5.93 ± 0.93	6.07 ± 0.15
Albumin (g dL^─1^)	4.47 ± 0.67	4.27 ± 0.15	3.93 ± 0.67	4.20 ± 0.46
Globulin (g dL^─1^)	1.73 ± 0.35	1.77 ± 0.65	2.00 ± 0.26	1.87 ± 0.31
Triglyceride (mg dL^─1^)	191.33 ± 7.57^b^	203.37 ± 5.51^ab^	211.33 ± 14.57^a^	203.67 ± 6.03^ab^
Glucose (mg dL^─1^)	104.33 ± 5.03 ^b^	109.33 ± 5.51^ab^	116.00 ± 7.00^ab^	119.67 ± 7.51^a^
GPT (U mL^─1^)	27.33 ± 5.86	28.67 ± 5.69	25.67 ± 2.52	29.00 ± 5.29
AST (U mL^─1^)	68.6 ± 2.08^a^	64.30 ± 2.23^b^	58.61 ± 3.46^b^	56.43 ± 2.31^b^
ALT (U mL^─1^)	27.32 ± 3.38	28.71 ± 3.28	25.72 ± 1.45	29.17 ± 3.05

AN_0_, AN_2_, AN_4_, and AN_6_ are diets supplemented with *A. platensis* nanoparticles at levels of 0, 2, 4, and 6 g kg^─1^ diet, respectively. GPT: Glutamic pyruvate transaminase (U mL^─1^), AST: Aspartate aminotransferase (U mL^─1^), ALT Alanine (U mL^─1^). The presented data are Means ± SD (*n = 3*). Different letters in the same row are significantly different (*p < 0.05*), while the absence of letters means no significant differences.
